# Intra- and Inter-Day Reliability of Inertial Loads with Cluster Sets When Performed during a Quarter Squat on a Flywheel Device

**DOI:** 10.3390/sports11060121

**Published:** 2023-06-19

**Authors:** Shane Ryan, Rodrigo Ramirez-Campillo, Declan Browne, Jeremy A. Moody, Paul J. Byrne

**Affiliations:** 1 Department of Health and Sport Sciences, South East Technological University, Kilkenny Road Campus, R93 V960 Carlow, Ireland; shane.ryan@setu.ie (S.R.); declan.browne@setu.ie (D.B.); 2 Exercise and Rehabilitation Sciences Institute, School of Physical Therapy, Faculty of Rehabilitation Sciences, Universidad Andres Bello, Santiago 7550196, Chile; rodrigo.ramirez@unab.cl; 3 Cardiff School of Sport and Health Sciences, Cardiff Metropolitan University, Cardiff CF5 2YB, UK; jmoody@cardiffmet.ac.uk

**Keywords:** eccentric overload, isoinertial, power, resistance training

## Abstract

The aims of this study were to (i) estimate the intra- and inter-day reliability of mean concentric (CON) and eccentric (ECC) power at different inertial loads during a flywheel quarter-squat using a cluster set approach and (ii) to determine the acute effect of internal and external attentional focus on mean power when performing the flywheel quarter squat. Twelve collegiate field sport male athletes (age 22.4 ± 3.2 years, weight 81.4 ± 10.3 kg, height 1.81 ± 0.06 m) attended four cluster set testing sessions separated by 7 days. Sessions consisted of 4 sets of 15 repetitions using 4 inertial loads (0.025, 0.050, 0.075, and 0.100 kg·m^2^). A cluster block consisted of 5 repetitions, including “momentum repetitions” (4 × 5 + 5 + 5). Mean power (MP), CON power, ECC power, and ECC overload were recorded for both internal and external attentional focus groups. The external instructional group attained familiarization after two flywheel sessions (ES = 0.03–0.15) with little volatility between performance measures (CV% = 3.39–9.22). The internal instructional group showed large differences in MP output from session 2 to session 3 for all loads (ES = 0.59–1.25). In conclusion, the flywheel cluster set approach is a reliable training modality for maintaining MP output during all repetitions.

## 1. Introduction

Flywheel iso-inertial training (FIT) has grown in popularity since its initial exploration by NASA to mitigate muscular atrophy due to the anti-gravitational environment faced by astronauts during space travel [1,2]. FIT utilizes the force generated during the concentric (CON) phase of a movement to wind a tether connected via a shaft to a rotating weighted flywheel disc. During exercise, the user accelerates the flywheel disc, exerting maximal force throughout the CON phase of the movement. As the tether winds, the device can store kinetic energy from the flywheel disc [3]. Immediately following the CON phase, the potential energy is at the highest level and can subsequently be used to enhance the FIT stimulus during the following eccentric (ECC) phase [4,5]. When FIT is performed efficiently and the necessary breaking action is delayed until the final third of the ECC movement, ECC overload can be achieved [6,7]. An ECC overload stimulus is when the ECC force is greater than the preceding CON force applied during an exercise [8]. To date, FIT research has reported improvements in explosive movements such as jumping [9,10], sprinting [11], and change of direction (COD) [6,12]. Moreover, previous studies have shown the positive effects of FIT on strength and power output [13,14]. However, there is minimal FIT research examining the role of familiarization and reliability of kinetic and kinematic measures during a quarter squat.

The familiarization period is widely considered a crucial component of FIT to stabilize flywheel kinematic and kinetic data to ensure that FIT has the desired training effect [15]. To the best of the authors’ knowledge, one study to date has sought to establish a familiarization protocol [7]. The concept of the familiarization protocol (2–4 sessions) prior to commencing FIT is part of the gross motor learning process required to acquire the skill that is creating and absorbing the ECC overload [16]. Furthermore, the familiarization protocol produced by Sabido et al. [7] consisted of 1 set of 10 repetitions using 4 inertial weighted discs (0.025, 0.050, 0.075, and 0.100 kg·m^2^). Sabido et al. [7] concluded that 2–3 sessions were necessary for familiarization, although improvements in performance were observed even into the fourth session. However, despite promising initial reports by Sabido et al. [7], it was noted that performance during the flywheel quarter-squat experienced various drop-offs in velocity and CON/ ECC output independent of inertial load used (e.g., repetition 5 using the 0.05 kg·m^2^). Therefore, as FIT is heavily focused on the generation of ECC overload, which is a subsequent result of an individual’s peak CON output capabilities, it would appear necessary to investigate a means of preventing this drop-off in CON velocity. Thus, developing a familiarization protocol employing a cluster set (CS) system may assist athletes in maintaining velocity and CON/ECC output in a FIT quarter squat [17].

Traditionally, resistance training (RT) follows a set configuration, the completion of all repetitions within a working set in a continuous fashion, with scheduled rest only present between subsequent sets [18]. This configuration is referred to as a “traditional set” (TS) [19]. This approach has reported positive adaptations among strength-related measures; however, it is associated with high levels of fatigue and metabolite accumulation [20]. Alternatively, in recent decades a training mode involving set-manipulation during RT has been investigated. First proposed by Roll and Omer [21], the “cluster set” (also called “rest-pause”) training method uses intra-set manipulation during subsequent repetitions [19,22]. Cluster training can be manipulated in various ways, usually pertaining to the inclusion of intra-set rest periods or manipulating the load during specific repetitions [20,23]. Haff et al. [23] demonstrated higher levels of power output and barbell velocity when comparing a traditional barbell set vs. a traditional CS approach with the same loads. Furthermore, Davies et al. [20] indicated that CS can achieve similar results as traditional RT; however, the cluster approach induces less systemic fatigue in the individual. Moreover, the reduction in fatigue has also reported improvements in movement quality, hence increasing the likelihood of greater performance during highly skilled multi-joint movements [24,25].

In the context of skill acquisition during FIT, a limitation of the literature is the lack of inclusion of coaching instructions within methodologies [17,26,27]. Many studies examining FIT provide no description of the attentional focus strategy used, commonly reporting “subjects were encouraged to perform CON/ECC phases as fast as possible” with no context surrounding the specific cues or to what extent it impacted the athlete’s performance [15,26]. A study by Branscheidt et al. [28] suggests that mastering a new movement requires a complexity of repetition and intensive practice. Furthermore, Russell et al. [29] suggest including a pre-practice phase including verbal coaching strategies, regarded as essential parts of the skill acquisition process. Based upon the limitations of the FIT literature and the research by Branscheidt et al. [28] and Russell et al. [29], investigating the effect of coaching instructions is warranted to maximize the effect of the familiarization protocol and to determine if there are differences between different types of instruction (i.e., internal versus external attentional focus instructions). Moreover, the CS offers intra-set rest periods through which instructional coaching cues can be re-emphasized and act as a complexity of repetition and intensive practice.

To date, one study has investigated the effects of familiarization on FIT kinetic and kinematic data in a quarter squat using a traditional repetition set system, which has been reviewed earlier in this section [7]. To the best of our knowledge, no previous studies have examined a familiarization phase using a flywheel CS training approach or estimated the intra- and inter-day reliability of employing a flywheel CS approach and the acute effect of internal and external attentional focus instructions in conjunction with a quarter squat. Thus, the aims of this study were (i) to estimate the intra- and inter-day reliability for these same measures with the same protocol and (ii) to determine the acute effect of internal and external attentional focus on mean power when performing the flywheel quarter squat. It was hypothesized that the intra-set rest periods would aid in the maintenance of performance measures and positively impact the skill acquisition process during the familiarization period.

## 2. Materials and Methods

### 2.1. Study Design

**Figure 1:**
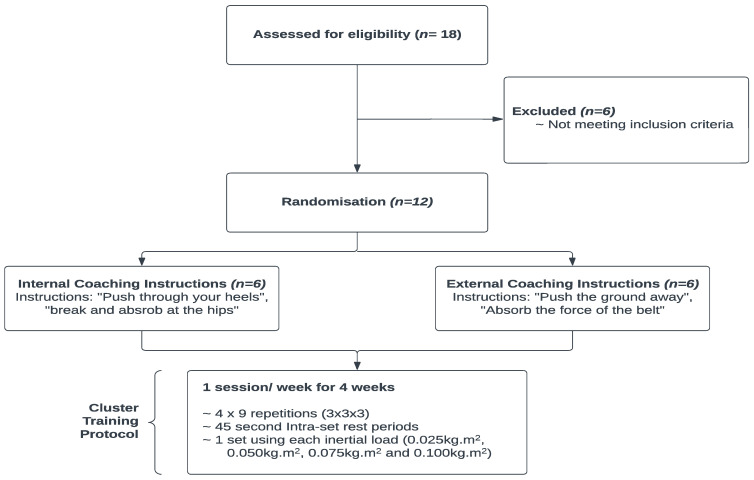
Testing procedure including specific coaching instructions and weekly flywheel training program.

This study applied an intra-day and inter-day design to estimate reliability during a CS flywheel protocol for the quarter squat. Additionally, a parallel group design was used to compare the acute responses between internal and external attentional focus verbal instructions on kinetic and kinematic measures using a CS (Figure 1).

### 2.2. Participants

Eighteen amateur male collegiate field sport (partaking in Gaelic football, hurling, and soccer) athletes (age 22.4 ± 3.2 years, weight 81.4 ± 10.3 kg, height 1.81 ± 0.06 m) volunteered to participate in this study. However, only twelve of these athletes completed the study due to missing a testing session or becoming infected with COVID-19. A sample size of convenience was used for the current study. Eligibility criteria dictated that participants be deemed as “resistance trained individuals” based upon training experience (2.0 ± years) and a relative one-repetition maximum (1 RM) lower body strength level (1.5 × body weight) for the half squat. Moreover, all participants were required to have no previous experience using a flywheel device, which may have influenced their ability to influence the familiarization process. Failure to meet these criteria meant participants were excluded from participation. At the time of testing, participants were tested during their off-season, which included reduced training schedules. Participants, on average, were training three times per week (one field session, two weight training sessions) at the time of participation. Participants were encouraged to continue their normal training while the study was ongoing. All participants attended testing sessions at the same time each week (±2 h) and were instructed to refrain from strenuous activity and any type of stimulant for the 48 h prior to testing sessions. All participants had no previous orthopaedic or musculoskeletal injuries to the lower extremities in the previous 6 months based upon medical screening. Prior to giving their informed consent to participate, a briefing of the testing procedures, benefits, and possible risks was presented to all participants. Experimental procedures were completed following the Declaration of Helsinki and approved by the South East Technological University research ethics board (Ethics Board Application Number: 281).

### 2.3. Famialirisation Protocol

This study used a randomized repeated measures design, whereby participants were randomly allocated into one of two instructional focus groups. Participants then underwent a repeated measures design where male field sport athletes completed 4 FIT testing sessions, separated by 7 days to determine inter-day reliability peak/mean power output and ECC overload [30]. Prior to partaking in the first testing session, Participants were divided into 2 groups of 6: internal focus group (*n* = 6) and external focus group (*n* = 6). During each visit to the laboratory, an athlete randomly completed 1 set of 15 repetitions (3 × 5 + 5 + 5) with each of the 4 inertial loads (0.025 kg·m^2^, 0.05 kg·m^2^, 0.075 kg·m^2^ and 0.100 kg·m^2^). Participants attended 4 testing sessions separated by 7 days. Each flywheel testing session began with a warm-up consisting of a 5-min self-paced jog, followed by 5 dynamic stretches (quadriceps, hamstrings, gluteal, adductors, and gastrocnemius). Each stretch was performed over 10 m for 14 repetitions per leg [31]. Thereafter, each participant performed a submaximal warm-up set of 10 repetitions using a 0.05 kg·m^2^ inertial load.

The FIT cluster protocol consisted of 4 sets of 15 repetitions (4 × 5 + 5 + 5) of the quarter-squat exercise performed on a flywheel device (kBox 4, Exxentric, AB TM, Bromma, Sweden). Each CS consisted of 3 individual 5-repetition cluster blocks (CB) within the full set. The first and second repetitions of each CB were used to increase velocity of the weighted disc, thus regarded as “momentum repetitions” and excluded from data analysis. Upon completion of each 5-repetition CB, intra-set rest periods of 45 s were taken [23]. Each subject was provided with a 4-min rest between completed sets (15 repetitions). Four inertial loads (0.025 kg·m^2^, 0.05 kg·m^2^, 0.075 kg·m^2^, and 0.100 kg·m^2^) were used throughout the study. The order of the inertial loads was performed in either ascending order from 0.025 kg·m^2^ to 0.100 kg·m^2^ or in descending order from 0.100 kg·m^2^ to 0.025 kg·m^2^, with the order being randomized. Each session consisted of 60 repetitions in total.

### 2.4. Mechanical Range of Motion

To standardize testing procedures, range of motion was limited from full knee extension at 180° to a FIT quarter-squat at a knee joint angle of 135° [7]. This was assessed using a goniometer (large goniometer, Cartwright fitness Ltd., Chester, UK), and a piece of tape and band were used as a marker for the individualized standard depth. During the CON phase of the exercise, participants were instructed to perform the movement as fast as possible with maximal intent, followed by a delay in the breaking action until the final third of the ECC phase [4]. Ankle extension was not allowed.

### 2.5. kMeter Application

The kMeter application (app) was used to assess the CON/ECC ratio of each load in addition to mean and peak power scores, which were recorded to assess drop-offs in performance. The kMeter app has been reported as a reliable measure of mean CON and ECC power outputs during FIT [32]. Moreover, Weakley et al. [32] used typical error of the estimate (TEE) to assess reliability, reporting >10% using all inertial loads. Thus, a limitation of the study may be the lack of ICC and CV% reported as a method of reliability. Variables used during data analysis were peak and mean concentric and eccentric power output. Familiarization was deemed to have been achieved when a subject performed two sessions in succession without significant difference in mean force output [7]. The ECC overload was calculated in both absolute (Nm = ECC peak force/CON peak force) and relative values (ECC peak force/100/CON peak force/100).

### 2.6. Coaching Instructions

During all testing sessions, participants in both attentional focus groups received either internal or external focus instructions to focus their lower body power production to absorb the ECC overload stimulus during the repetitions. During the FIT cluster quarter-squat exercise, the internal focus instruction group received the following instructions: “Break and absorb at your hips” and “Push through your heels”. The external focus instruction group received the following instructions: “Absorb the force of the belt” and “Push the ground away”. All coaching instructions were administered prior to the exercise and then reiterated during the 45-s intra-set rest periods.

### 2.7. Statistical Analysis

After data normality assumptions were verified with the Shapiro-Wilks test, means and standard deviations were calculated for all measures. Intra and inter-day reliability was assessed using an intra-class correlation coefficient (3.1, 2-way mixed model with consistency and average effect measure) and 95% confidence intervals. Average measures from this ICC model and Cronbach’s alpha were also reported for individual and mean power output during cluster sets. The interpretation of ICC values was poor (0.00–0.49), moderate (0.50–0.69), high (0.70–0.89), and very high (≥0.9) [33]. Absolute reliability was assessed using coefficient of variance (CV%) and standard error of measurement (SEM). The CV was calculated as standard deviation/mean × 100 with a cut-off point for acceptability set at 10% [34]. The SEM was calculated as SEM = SD × √(1-ICC) [35]. A Shapiro-Wilks test of normality was run to assess distribution among inertial loads’ mean power (MP) output. A repeated measures analysis of variance (ANOVA) with a Bonferroni post-hoc was used to assess the internal and external 0.025 kg·m^2^ and 0.050 kg·m^2^ inertial loads. Friedman tests were conducted when comparing non-parametric testing days within the inertial loads of 0.075 kg·m^2^ and 0.100 kg·m^2^. Statistical significance was set at *p* ≤ 0.05. To determine the effect size (ES), Cohen’s d was calculated, where the effect size (d) is interpreted as <0.2 (trivial), 0.2–0.5 (small), >0.5–0.8 (moderate), and >0.8 (large) [36]. The ES was used to estimate where in the familiarization process the greatest learning effect took place between subsequent sessions (day 1 vs. day 2, day 2 vs. day 3, day 3 vs. day 4). The smallest worthwhile change (SWC) was calculated to assess meaningful changes between measures at 0.5× the standard deviation for each CS. Typical error was used to assess the smallest worthwhile change and interpreted as marginal (TE < SWC) or good (TE > SWC) [37]. Statistical analyses were carried out using IBM SPSS Version 27 (SPSS, Inc., Chicago, IL, USA).

## 3. Results

The means and standard deviations of each individual inertial load across separate testing days are displayed for both internal and external instructional groups in Table 1.

Intra-day reliability scores for MP output using each inertial load (0.025, 0.050, 0.075, and 0.10 kg·m^2^) are displayed in Table 2. Inter-day reliability scores for MP output using each inertial load (0.025, 0.050, 0.075, and 0.10 kg·m^2^) are displayed in Table 3. Independent of the inertial load used, each flywheel CS reported excellent reliability with the successive days which followed. Cohen’s d effect size (ES) indicated that the external group experienced a large change between day 1 and day 2 for each inertial load; thereafter, the following days saw trivial changes on all but one inertial load (0.025 kg·m^2^, during day 4), whereas the internal group reported a moderate ES (<0.5) using the 0.025 kg·m^2^ and 0.100 kg·m^2^ inertial loads. In addition, a large change (ES ≤ 0.8) was reported between day 2 and day 3 using the 0.050 kg·m^2^ and 0.075 kg·m^2^ inertial loads.

**Figure 2:**
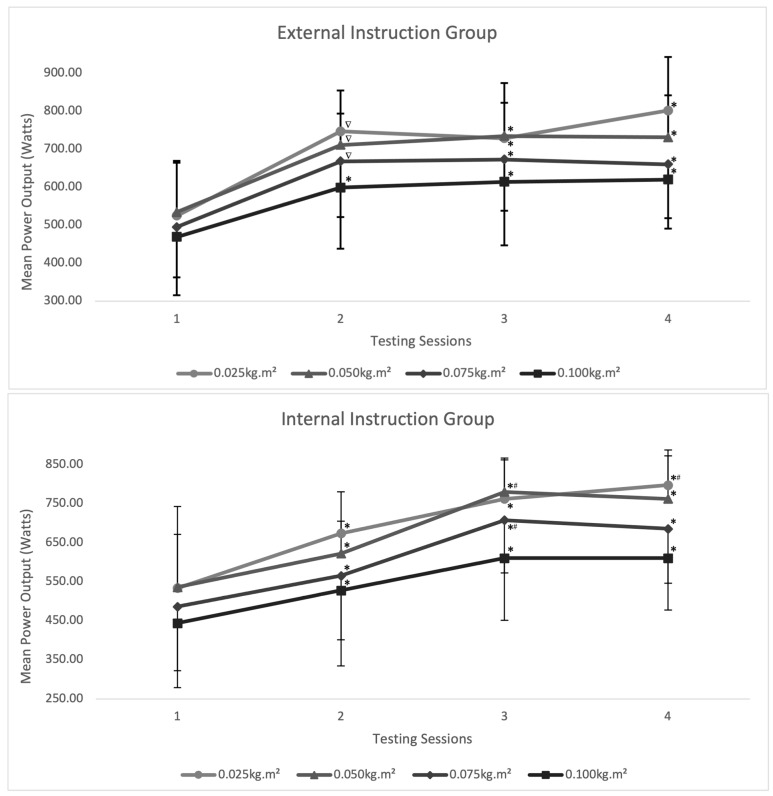
Inter-day inter-set mean power (MP) output (Watts) by inertial load during the flywheel quarter squat exercise from sessions 1 to 4. * = significantly greater than day 1 (*p* ≤ 0.05); ∇ = significantly greater than day 1 (*p* ≤ 0.001); # = significantly greater than day 2 (*p* ≤ 0.001).

Independent of the instructional cue and inertial load used, all days showed a significant difference (*p* ≤ 0.05) between day 1 MP output and all other testing days. The external instructional group showed no significant difference in MP output after day 2, while the internal instructional group showed a significant difference (*p* ≤ 0.05) between day 2 and day 3 power outputs when using then 0.050 kg·m^2^ and 0.075 kg·m^2^ inertial loads. All other inertial loads displayed no significant differences between day 2 MP output and day 3 or day 4 (see Figure 2).

It should be noted that during the testing days, the best CB varied among the 3 (5-repetition) sets, albeit no significant differences were noted between sets (CB1, CB2, CB3). Intra-set repetitions for both CON/ ECC output independent of inertial load were analyzed for decrements in output due to muscular fatigue during the CS. When compared to the best repetition, the internal group showed no significant differences between the peak power output and minimum power output repetition during all sets and inertial loads. Based on ES values, no power decrements were observed during any of the internal cluster sets. Similarly, the external group reported almost indistinguishable consistency throughout intra-set CON/ ECC outputs, independent of the inertial load used (see Table 4).

The external group reported a significant difference (*p* = 0.006) between the first repetition and the best repetition during the 0.025 kg·m^2^ inertial load. Furthermore, it was observed that the peak power repetition was commonly observed in the later CBs, between the 6th and 9th repetition, or in CB2 or CB3. As familiarization was deemed for the external group, an observation was noted that the efficiency to produce ECC overload increased with inertial load (see Table 5). Independent of the instructional coaching group, it was reported that the 0.05 kg·m^2^ inertial load produced the greatest CON and ECC power output.

Table 5 and Table 6 show the influence of inertial load on CON/ECC power output. In addition, the ratio of ECC overload is displayed for both instructional groups. Independent of the instructional group, CON power was substantially greater with the 0.025 kg·m^2^ inertial load, albeit the 0.050 kg·m^2^ inertial load resulted in similar levels during day 3 and day 4 output measures. In addition, the 0.050 kg·m^2^ produced greater CON output than the 0.075 kg·m^2^ and the 0.075 kg·m^2^ resulted in substantially greater CON output than the 0.100 kg·m^2^ inertial load. Contrary to FIT literature, the 0.025 kg·m^2^ inertial load reported the greatest ECC output during session 1 in comparison to all other inertial loads. During this initial stage, it was theorized that skill acquisition was more difficult to attain using heavier inertial loads. Moreover, post-session 1, there was a noticeable difference between instructional groups, as the internal instructional group reported significantly lower ECC overload using the 0.100 kg·m^2^ inertial load during sessions 3 and 4 (see Table 5 and Table 6).

## 4. Discussion

The aims of this study were to (i) estimate the intra- and inter-day reliability for these same measures with the same protocol and (ii) determine the acute effect of internal and external attentional focus on mean power when performing the flywheel quarter squat. Our findings suggest that stability of CON and ECC power output was obtained after the second familiarization day, albeit the skill acquisition period is largely influenced by the specific instructional focus used during this process. In this sense, both internal versus external coaching instructions produced similar end values for MP output, although a large difference was reported in MP output during the second session, independent of the inertial load used (see Table 1). Furthermore, a significant difference was noted between instructional groups in the context of the rate of time taken to reach skill acquisition using the 0.05 kg·m^2^ inertial load (*p* = 0.046) and 0.075 kg·m^2^ inertial load (*p* = 0.017).

Previous FIT research reported good reliability scores for mean and peak power output during the flywheel quarter-squat exercise (ICC = 0.79–0.93 and CV% = 7.5–13.2). Consequently, when compared to the reliability scores of Sabido et al. [10], the present study using the FIT intra-set rest periods reported excellent levels of reliability for both CON and ECC power output, in addition to power maintenance across both instructional groups. Moreover, when comparing both instructional groups in relation to aiding in the skill acquisition process, this study reported highly reliable ICC and CV% values for both groups (external group ICC = 0.88–0.99 and CV% = 3.39–11.26; internal group ICC = 0.88–0.99 and CV% = 3.56–10.10) (see Table 2). Furthermore, ES values were used to estimate where in the familiarization process the greatest learning effect took place between instructional groups. The external instructional group reported very large ES values (0.96–1.24) between the initial testing session and day 2; thereafter, trivial ES values (<0.15) were reported in all testing sessions apart from the 0.025 kg·m^2^ inertial load between sessions 3 and 4. Alternatively, the internal instructional coaching group reported moderate (ES = 0.68) and large (ES = 0.90) effects during the initial day and second testing day, following which, large–very large ES were reported between day 2 and 3 (0.59–1.25) (see Table 4). Moreover, these reports suggest that incorporating external coaching instructions will aid in the gross motor learning process during FIT.

The familiarization period is considered necessary to stabilize flywheel kinematic and kinetic data to ensure that FIT has the desired training effect [15]. When considering the stability of velocity throughout intra-day performance output, the previous FIT literature commonly reports a decrement in mean and peak power output during sets of ≥ 6 repetitions [7,38]. In comparison, excluding the first repetition using the 0.025 kg·m^2^ inertial load for the external group, the CS method was effective in maintaining maximal CON and ECC power output throughout all individual repetitions (in both instructional groups). Limiting the inter-day variance in the familiarization period is a vital component in optimizing the familiarization process [8,38]. Moreover, as expected, the first testing session reported the greatest variance in CV% (internal group = 8.44–15.41%; external group = 7.37–16.14%). This report aligns with similar inter-day FIT reliability literature stating that a minimum of 2–3 sessions were required to observe stability in FIT output, depending on the exercise involved [7,39]. Furthermore, Beato et al. [38] reported excellent inter-day reliability over 2 testing sessions separated by 1 week, although an additional familiarization session was included 72 h prior to the initial session (3 sessions in total).

An athlete’s ability to produce high muscular power has been linked to success on the field, and power output during training influences neuromuscular adaptations post-training [23,40]. Based on previous research, maximal power production is dependent on two central components, including an athlete’s ability to produce high levels of force rapidly and prompt high contraction velocities [23,41]. It is encouraging to compare the present fatigue manipulation findings with previous research by Tufano et al. [42], who reported no significant difference between mean and peak CON force output between TD and CS during a back squat protocol, albeit a significant difference was noted in the mean and peaks for CON power and velocity using the CS approach [42]. Moreover, it has been reported in previous research by Ritti-Dias et al. [43] that the traditional barbell back squat technique takes a minimum of two sessions to achieve stability for 1RM in untrained individuals, suggesting similarity to FIT modalities.

This study encountered a few limitations. The primary limitation is the limited concluding sample size (*n* = 12). Resource constraints were the determining factor behind this low sample number due to restrictions in place from the COVID-19 pandemic [44]. Moreover, COVID-19 was an obstacle when recruiting sufficient participants and also contributed to the high rate of dropouts. Initially, 18 suitable participants were recruited but due to high drop-out incidences, concluded with 12 (6 participants per instructional focus group). Furthermore, due to constraints outside of the researchers’ control, the sample size was estimated at a sample of convenience. Thus, due to the low sample size this study concluded with, this study’s findings must be carefully interpreted. Moreover, another possible limitation of the flywheel cluster approach may be the lack of maximal repetitions individuals are exposed to per cluster set once peak velocity has been reached (3 repetitions). Furthermore, in a training setting, the work/ rest ratio, including the 45-s intra-set rest period, may not be widely accepted through training logistics. This is a dilemma through fatigue manipulation and optimal loading parameters when training for maximal power production.

## 5. Conclusions

In conclusion, this is the first study to investigate the inter and intra-day reliability of a FIT quarter-squat protocol using cluster sets. The use of intra-set rest periods reported excellent reliability (ICC and CV%) for both peak and mean CON and ECC power output and ECC overload measures. Our findings suggest that FIT cluster training may be an effective alternative training mode to incorporate into FIT strategies, especially when peak CON and ECC power output are the desired training goal. Future research should examine the optimal “intra-set” rest period for performance while maintaining maximal velocity. The findings of the current study proved that a significant improvement in performance can be obtained by utilizing correct instructional cues. It is important for practitioners to utilize external coaching instructions during the process as significant differences were noted between instructional groups and participants’ ability to achieve ECC overload.

## Figures and Tables

**Table 1:** Mean power output (W; mean ± standard deviation) by inertial load and familiarization testing day for the internal and external attentional focus groups.

Internal Group				
Inertial Load	Day 1	Day 2	Day 3	Day 4
0.025 kg·m^2^	533 ± 209	673 ± 135	762 ± 164	797 ± 165
0.050 kg·m^2^	536 ± 107	622 ± 83	780 ± 164	762 ± 193
0.075 kg·m^2^	486 ± 100	565 ± 87	707 ± 135	685 ± 159
0.10 kg·m^2^	443 ± 90	527 ± 110	610 ± 139	610 ± 133
**External Group**				
**Inertial Load**	**Day 1**	**Day 2**	**Day 3**	**Day 4**
0.025 kg·m^2^	524 ± 139	746 ± 211	728 ± 133	801 ± 154
0.050 kg·m^2^	533 ± 107	710 ± 176	734 ± 147	731 ± 161
0.075 kg·m^2^	495 ± 145	667 ± 149	672 ± 135	659 ± 167
0.10 kg·m^2^	469 ± 141	598 ± 128	613 ± 142	620 ± 129

**Table 2:** Intra-day reliability statistics reporting Cronbach’s α and ICC single and average measure and 95% confidence intervals (CI) both single and average measures for 0.025, 0.050, 0.075, and 0.100 kg·m^2^ flywheel squat mean power outputs between complete sets over the four familiarisation testing sessions for the internal and external attentional focus groups.

Internal Group					
Inertial Load	Cronbach’s α	Single Measure	95% CI	Average Measure	95% CI
0.025 kg·m^2^ (Day 1)	0.93	0.81	0.42–0.97	0.93	0.68–0.99
0.025 kg·m^2^ (Day 2)	0.93	0.81	0.42–0.97	0.93	0.69–0.99
0.025 kg·m^2^ (Day 3)	0.88	0.70	0.24–0.95	0.88	0.48–0.98
0.025 kg·m^2^ (Day 4)	0.95	0.86	0.55–0.98	0.95	0.79–0.99
0.050 kg·m^2^ (Day 1)	0.91	0.77	0.36–0.96	0.91	0.62–0.99
0.050 kg·m^2^ (Day 2)	0.91	0.77	0.35–0.96	0.91	0.62–0.99
0.050 kg·m^2^ (Day 3)	0.98	0.95	0.80–0.99	0.98	0.92–1.00
0.050 kg·m^2^ (Day 4)	0.99	0.97	0.87–1.00	0.99	0.95–1.00
0.075 kg·m^2^ (Day 1)	0.97	0.92	0.72–0.99	0.97	0.88–1.00
0.075 kg·m^2^ (Day 2)	0.94	0.84	0.49–0.97	0.94	0.74–0.99
0.075 kg·m^2^ (Day 3)	0.96	0.90	0.65–0.98	0.96	0.85–1.00
0.075 kg·m^2^ (Day 4)	0.98	0.93	0.74–0.99	0.98	0.90–0.99
0.100 kg·m^2^ (Day 1)	0.88	0.72	0.26–0.95	0.88	0.51–0.98
0.100 kg·m^2^ (Day 2)	0.98	0.95	0.82–0.99	0.98	0.93–1.00
0.100 kg·m^2^ (Day 3)	0.92	0.80	0.40–0.97	0.92	0.67–0.99
0.100 kg·m^2^ (Day 4)	0.96	0.89	0.61–0.98	0.96	0.83–0.99
**External Group**					
**Measures**	**Cronbach’s α**	**Single Measure**	**95% CI**	**Average Measure**	**95% CI**
0.025 kg·m^2^ (Day 1)	0.93	0.82	0.48–0.97	0.93	0.71–0.99
0.025 kg·m^2^ (Day 2)	0.96	0.90	0.65–0.98	0.96	0.85–1.00
0.025 kg·m^2^ (Day 3)	0.96	0.89	0.61–0.98	0.96	0.82–0.99
0.025 kg·m^2^ (Day 4)	0.88	0.70	0.23–0.95	0.88	0.47–0.98
0.050 kg·m^2^ (Day 1)	0.94	0.84	0.49–0.97	0.94	0.74–0.99
0.050 kg·m^2^ (Day 2)	0.99	0.97	0.88–1.00	0.99	0.96–1.00
0.050 kg·m^2^ (Day 3)	0.97	0.92	0.72–0.99	0.97	0.89–1.00
0.050 kg·m^2^ (Day 4)	0.99	0.98	0.93–1.00	0.99	0.98–1.00
0.075 kg·m^2^ (Day 1)	0.87	0.70	0.22–0.94	0.87	0.46–0.98
0.075 kg·m^2^ (Day 2)	0.97	0.92	0.71–0.99	0.97	0.88–1.00
0.075 kg·m^2^ (Day 3)	0.98	0.96	0.83–0.99	0.98	0.93–1.00
0.075 kg·m^2^ (Day 4)	0.98	0.94	0.78–0.99	0.98	0.91–1.00
0.100 kg·m^2^ (Day 1)	0.98	0.95	0.82–0.99	0.98	0.93–1.00
0.100 kg·m^2^ (Day 2)	0.94	0.85	0.51–0.98	0.94	0.76–0.99
0.100 kg·m^2^ (Day 3)	0.99	0.97	0.88–100	0.99	0.96–1.00
0.100 kg·m^2^ (Day 4)	0.98	0.95	0.82–0.99	0.98	0.93–1.00

**Table 3:** Inter-day reliability statistics reporting Cronbach’s α and ICC single and average measures and 95% confidence intervals (CI) for 0.025, 0.050, 0.075, and 0.100 kg·m^2^ flywheel squat mean power outputs between complete sets over the four familiarisation testing sessions for the internal and external attentional focus groups.

Intraclass Correlation Coefficient (ICC) Internal Group					
Measures	Cronbach’s α	Single Measure	95% CI	Average Measure	95% CI
0.025 kg·m^2^ (Day 1–2)	0.30	0.18	−0.30–0.59	0.30	−0.86–0.74
0.025 kg·m^2^ (Day 2–3)	0.85	0.73	0.42–0.89	0.85	0.59–0.94
0.025 kg·m^2^ (Day 3–4)	0.79	0.65	0.28–0.85	0.79	0.43–0.92
0.050 kg·m^2^ (Day 1–2)	0.32	0.19	−0.29–0.59	0.32	−0.83–0.74
0.050 kg·m^2^ (Day 2–3)	0.95	0.91	0.78–0.97	0.95	0.88–0.98
0.050 kg·m^2^ (Day 3–4)	0.95	0.91	0.77–0.96	0.95	0.87–0.96
0.075 kg·m^2^ (Day 1–2)	0.61	0.44	−0.03–0.74	0.61	−0.53–0.85
0.075 kg·m^2^ (Day 2–3)	0.93	0.87	0.69–0.95	0.93	0.82–0.98
0.075 kg·m^2^ (Day 3–4)	0.97	0.95	0.87–0.98	0.97	0.93–0.99
0.100 kg·m^2^ (Day 1–2)	0.78	0.64	0.25–0.85	0.78	0.40–0.92
0.100 kg·m^2^ (Day 2–3)	0.89	0.80	0.53–0.92	0.89	0.70–0.96
0.100 kg·m^2^ (Day 3–4)	0.95	0.91	0.77–0.96	0.95	0.87–0.98
**External Group**					
**Measures**	**Cronbach’s α**	**Single Measure**	**95% CI**	**Average Measure**	**95% CI**
0.025 kg·m^2^ (Day 1–2)	0.79	0.66	0.28–0.86	0.79	0.44–0.92
0.025 kg·m^2^ (Day 2–3)	0.80	0.67	0.30–0.86	0.80	0.46–0.93
0.025 kg·m^2^ (Day 3–4)	0.83	0.70	0.36–0.88	0.83	0.53–0.94
0.050 kg·m^2^ (Day 1–2)	0.59	0.42	−0.05–0.73	0.59	−0.10–0.85
0.050 kg·m^2^ (Day 2–3)	0.78	0.64	0.25–0.85	0.78	0.40–0.92
0.050 kg·m^2^ (Day 3–4)	0.95	0.90	0.75–0.96	0.95	0.86–0.98
0.075 kg·m^2^ (Day 1–2)	0.87	0.77	0.48–0.91	0.87	0.65–0.95
0.075 kg·m^2^ (Day 2–3)	0.70	0.54	0.11–0.80	0.70	0.20–0.89
0.075 kg·m^2^ (Day 3–4)	0.94	0.88	0.72–0.96	0.94	0.83–0.98
0.100 kg·m^2^ (Day 1–2)	0.68	0.51	0.08–0.79	0.68	0.14–0.88
0.100 kg·m^2^ (Day 2–3)	0.59	0.42	−0.05–0.73	0.59	−0.11–0.85
0.100 kg·m^2^ (Day 3–4)	0.95	0.91	0.78–0.97	0.95	0.87–0.98

**Table 4:** Cohen’s D effect size (ES), Coefficient of variance (CV%), Typical Error (TE), and standard error of the measurement (SEM) reported in Watts and smallest worthwhile change (SWC) at 0.5 for each inertial load in successive day for the internal and external attentional focus groups.

Internal Group						
Measures	(ES)	CV (%)	TE	SWC0.5 (Watts)	SEM	Interpretation
0.025 kg·m^2^ (Day 1)	(D1–D2)–0.68	15.51	43.71	52.72	49.42	Good
0.025 kg·m^2^ (Day 2)	(D2–D3)–0.59	10.10	26.63	32.11	31.88	Good
0.025 kg·m^2^ (Day 3)	(D3–D4)–0.21	8.99	37.50	45.23	38.86	Good
0.025 kg·m^2^ (Day 4)		7.81	23.22	28.00	39.03	Good
0.050 kg·m^2^ (Day 1)	(D1–D2)–0.90	9.44	20.12	24.27	25.30	Good
0.050 kg·m^2^ (Day 2)	(D2–D3)–1.21	5.43	16.42	19.80	19.71	Good
0.050 kg·m^2^ (Day 3)	(D3–D4)–0.10	4.69	16.18	19.51	38.84	Good
0.050 kg·m^2^ (Day 4)		3.56	11.20	13.50	45.70	Good
0.075 kg·m^2^ (Day 1)	(D1–D2)–0.84	10.56	10.32	12.44	23.62	Good
0.075 kg·m^2^ (Day 2)	(D2–D3)–1.25	5.97	13.45	16.22	20.66	Good
0.075 kg·m^2^ (Day 3)	(D3–D4)–0.15	4.91	18.57	22.39	31.83	Good
0.075 kg·m^2^ (Day 4)		5.49	14.11	17.02	37.67	Good
0.100 kg·m^2^ (Day 1)	(D1–D2)–0.83	8.44	18.58	22.41	21.34	Good
0.100 kg·m^2^ (Day 2)	(D2–D3)–0.66	6.93	11.15	13.44	26.00	Good
0.100 kg·m^2^ (Day 3)	(D3–D4)–0.00	5.55	20.40	24.60	32.78	Good
0.100 kg·m^2^ (Day 4)		5.47	15.09	18.20	31.43	Good
**External Group**						
**Measures**	**(ES)**	**CV (%)**	**TE**	**SWC0.5 (Watts)**	**SEM**	**Interpretation**
0.025 kg·m^2^ (Day 1)	(D1–D2)–1.24	12.65	26.00	31.36	32.86	Good
0.025 kg·m^2^ (Day 2)	(D2–D3)–0.14	11.26	36.72	44.28	49.91	Good
0.025 kg·m^2^ (Day 3)	(D3–D4)–0.51	6.60	20.17	24.32	31.49	Good
0.025 kg·m^2^ (Day 4)		9.22	30.59	36.90	36.30	Good
0.050 kg·m^2^ (Day 1)	(D1–D2)–1.21	9.63	20.06	24.20	25.42	Good
0.050 kg·m^2^ (Day 2)	(D2–D3)–0.15	4.61	12.46	15.03	41.57	Good
0.050 kg·m^2^ (Day 3)	(D3–D4)–0.02	4.49	14.45	17.42	34.71	Good
0.050 kg·m^2^ (Day 4)		3.39	8.86	10.68	37.96	Good
0.075 kg·m^2^ (Day 1)	(D1–D2)–1.17	16.14	29.58	35.67	34.21	Good
0.075 kg·m^2^ (Day 2)	(D2–D3)–0.03	6.25	15.97	19.26	35.21	Good
0.075 kg·m^2^ (Day 3)	(D3–D4)–0.08	3.71	10.48	12.63	31.83	Good
0.075 kg·m^2^ (Day 4)		5.67	14.60	17.61	39.43	Good
0.100 kg·m^2^ (Day 1)	(D1–D2)–0.96	7.37	13.61	16.42	33.25	Good
0.100 kg·m^2^ (Day 2)	(D2–D3)–0.12	8.87	21.15	25.51	30.18	Good
0.100 kg·m^2^ (Day 3)	(D3–D4)–0.05	3.51	9.07	10.94	33.55	Good
0.100 kg·m^2^ (Day 4)		5.12	12.92	15.59	30.48	Good

**Table 5:** External group (*n*= 6). Concentric power, eccentric power, and eccentric/concentric ratio by inertial load and testing day.

Variable	Day 1	Day 2	Day 3	Day 4
Pcon 0.025 kg·m^2^	961.67 ± 227.76	1312.72 ± 299.86 *	1313.71 ± 225.41 *	1375.39 ± 217.51 *
Pecc 0.025 kg·m^2^	942.28 ± 214.14	1224 ± 403.05 *	1258.39 ± 296.34 *	1377.17 ± 218.65 *
Ratio 0.025 kg·m^2^	0.99 ± 0.11	0.92 ± 0.13	0.96 ± 0.14	1.01 ± 0.12
Pcon 0.050 kg·m^2^	942.50 ± 160.26	1256.61 ± 268.72 *	1332.89 ± 270.81 *	1282.67 ± 270.95 *
Pecc 0.050 kg·m^2^	934.83 ± 182.25	1315.17 ± 401.52 *	1468.11 ± 383.32 *	1474.06 ± 390.82 *
Ratio 0.050 kg·m^2^	1.00 ± 0.11	1.04 ± 0.16	1.11 ± 0.20	1.16 ± 0.23
Pcon 0.075 kg·m^2^	894.83 ± 256.21	1194.94 ± 256.40 *	1224.28 ± 251.58 *	1159 ± 284.20 *
Pecc 0.075 kg·m^2^	938.94 ± 308.49	1316.39 ± 351.16 *	1399.06 ± 356.28 *	1395.50 ± 465.56 *
Ratio 0.075 kg·m^2^	1.05 ± 0.10	1.10 ± 0.16	1.15 ± 0.20	1.21 ± 0.28
Pcon 0.100 kg·m^2^	819.11 ± 223.28	1094.89 ± 226.84 *	1106.33 ± 296.34 *	1085.61 ± 217.42 *
Pecc 0.100 kg·m^2^	886.17 ± 268.01	1248.94 ± 293.82 *	1263.50 ± 343.13 *	1335.94 ± 385.72 *
Ratio 0.100 kg·m_2_	1.08 ± 0.12	1.15 ± 0.16	1.15 ± 0.23	1.24 ± 0.32

P_con_ = peak concentric power output, P_ecc_ = peak eccentric power output, Ratio = eccentric overload ratio calculated by P_ecc_/P_con_ represented for each inertial load. * = significantly greater than day 1 (*p* ≤ 0.05).

**Table 6:** Internal group (*n* = 6). Concentric power, eccentric power, and eccentric/concentric ratio by inertial load and testing day.

Variable	Day 1	Day 2	Day 3	Day 4
Pcon 0.025 kg·m^2^	986.06 ± 364.41	1196 ± 210.55 *	1324.11 ± 287.02 *	1392.67 ± 311.23 *#
Pecc 0.025 kg·m^2^	1016.22 ± 334.95	1157.83 ± 233.66	1225.39 ± 250.41 *	1287.33 ± 243.01 *
Ratio 0.025 kg·m^2^	1.07 ± 0.15	0.97 ± 0.10	0.93 ± 0.10	0.94 ± 0.11
Pcon 0.050 kg·m^2^	945 ± 195.81	1109.83 ± 205.12 *	1363.17 ± 320.75 *#	1345.78 ± 372.30 *#
Pecc 0.050 kg·m^2^	976.50 ± 251.64	1184.06 ± 246.66 *	1372.11 ± 287.06 *#	1338.22 ± 342.30 *
Ratio 0.050 kg·m^2^	1.03 ± 0.12	1.07 ± 0.08	1.02 ± 0.10	1.00 ± 0.09
Pcon 0.075 kg·m^2^	870.28 ± 181.11	1021.83 ± 218.18 *	1248.67 ± 257.66 *#	1241.78 ± 331.41 *#
Pecc 0.075 kg·m^2^	940.83 ± 198.55	1133.56 ± 329.41 *	1318.28 ± 268.27 *	1306.72 ± 4393.68 *
Ratio 0.075 kg·m^2^	1.08 ± 0.10	1.10 ± 0.13	1.06 ± 0.08	1.05 ± 0.17
Pcon 0.100 kg·m^2^	824.17 ± 178.72	947.61 ± 245.07	1117.28 ± 302.25 *	1107.94 ± 286.99 *
Pecc 0.100 kg·m^2^	865.67 ± 205.75	1075.56 ± 294.79 *	1194.89 ± 332.85 *	1208.67 ± 352.85 *
Ratio 0.100 kg·m^2^	1.05 ± 0.05	1.14 ± 0.11	1.07 ± 0.09	1.09 ± 0.21

P_con_ = peak concentric power output, P_ecc_ = peak eccentric power output, Ratio = eccentric overload ratio calculated by P_ecc_/P_con_ represented for each inertial load. * = significantly greater than day 1 (*p* ≤ 0.05); # = significantly greater than day 2 (*p* ≤ 0.05).

## Data Availability

The data presented in this study are available upon request from the corresponding author.
